# Probing the Molecular Mechanism of Human Soluble Guanylate Cyclase Activation by NO *in vitro* and *in vivo*

**DOI:** 10.1038/srep43112

**Published:** 2017-02-23

**Authors:** Jie Pan, Hong Yuan, Xiaoxue Zhang, Huijuan Zhang, Qiming Xu, Yajun Zhou, Li Tan, Shingo Nagawa, Zhong-Xian Huang, Xiangshi Tan

**Affiliations:** 1 Department of Chemistry & Shanghai Key laboratory of Chemical Biology for Protein Research, Fudan University, Shanghai 200433, China; 2 Institutes of Biomedical Sciences, Fudan University, Shanghai 200433, China; 3 Shanghai Center for Plant Stress Biology, Shanghai Institutes for Biological Sciences, Chinese Academy of Sciences, Shanghai 200433, China

## Abstract

Soluble guanylate cyclase (sGC) is a heme-containing metalloprotein in NO-sGC-cGMP signaling. NO binds to the heme of sGC to catalyze the synthesis of the second messenger cGMP, which plays a critical role in several physiological processes. However, the molecular mechanism for sGC to mediate the NO signaling remains unclear. Here fluorophore FlAsH-EDT_2_ and fluorescent proteins were employed to study the NO-induced sGC activation. FlAsH-EDT_2_ labeling study revealed that NO binding to the H-NOX domain of sGC increased the distance between H-NOX and PAS domain and the separation between H-NOX and coiled-coil domain. The heme pocket conformation changed from “closed” to “open” upon NO binding. In addition, the NO-induced conformational change of sGC was firstly investigated *in vivo* through fluorescence lifetime imaging microscopy. The results both *in vitro and in vivo* indicated the conformational change of the catalytic domain of sGC from “open” to “closed” upon NO binding. NO binding to the heme of H-NOX domain caused breaking of Fe-N coordination bond, initiated the domain moving and conformational change, induced the allosteric effect of sGC to trigger the NO-signaling from H-NOX *via* PAS & coiled-coil to the catalytic domain, and ultimately stimulates the cyclase activity of sGC.

The diatomic gas nitric oxide (NO) is an essential signaling molecule in biology. NO signaling controls several physiological processes including vasodilation, neurotransmission and platelet aggregation^1^,^2^,^3^,^4^,^5^. Soluble guanylate cyclase (sGC) is a primary receptor of NO^6^. When activated, sGC catalyzes the conversion of substrate GTP to cGMP. cGMP, as a second messenger, triggers the downstream signaling cascades, including ion-gated channels, phosphodiesterases (PDEs) and cGMP-dependent protein kinases. Impairment of the NO-sGC-cGMP signaling has been linked to heart disease, hypertension, stroke, neurodegeneration and erectile dysfunction^7^,^8^. Therefore, sGC is a therapeutic drug target for treating diseases by improving the NO-sGC-cGMP signaling^9^,^10^.

The isoform α1β1 is ubiquitously distributed in cytosolic fractions of tissues, while α2β1 is mainly in brain. The most commonly studied and predominant sGC isoform is a Heme-containing heterodimeric enzyme composed of α1 and β1 subunits. Each subunit contains four domains: an N-terminal heme-NO/O_2_-binding (H-NOX) domain, a Per/Arnt/Sim (PAS) domain, a helical (CC) domain and a C-terminal catalytic domain^1^. However, the crystal structure of the human sGC holo-enzyme remains unknown, although one human sGC domain structure (catalytic domain) and homologies of other domains have been characterized by crystallography^11^,^12^,^13^,^14^,^15^. The H-NOX domain of β1 subunit binds heme through His105 and senses NO^11^,^16^, but the corresponding domain (pseudo-H-NOX) of α1 subunit does not bind heme and its function remains unclear. Both the PAS and CC domains of α1 and β1 subunits are involved in the heterodimer formation, cyclase activation regulation and signaling transmission^13^,^15^,^17^,^18^. The C-terminal catalytic domain constitutes the active site formed at the interface between α1 and β1 subunits^14^,^19^. Recently, the higher-order domain architecture of the sGC heterodimer have been reconstructed through diverse approaches, including small-angle X-ray scattering (SAXS), Hydrogen/deuterium exchange mass spectrometry (HDX-MS), and single-particle electron microscopy^20^,^21^,^22^. These studies have revealed that the inter-domain interactions are responsible for the NO-signaling from the H-NOX domain to the catalytic domain. Besides, the heme-binding H-NOX domain interacts directly with PAS domain, which allows small-scale changes in the H-NOX domain to be quickly sensed by the abutting PAS domain. The flexible PAS-helical linker and the helical-catalytic domain linker are involved in the NO-signaling transmitting. Behrends *et al*. confirmed that the close proximity of H-NOX domains and the catalytic domain using the fluorescent fusion proteins by FRET analysis^23^. Besides, Marletta *et al*. also indicated the similar results through HDX-MS studies^21^.

The molecular mechanism for NO-induced sGC activation remains a central question in NO-sGC-cGMP signaling. NO binding to the ferrous heme of sGC leads to the formation of a penta-coordinated Fe-NO complex through the dissociation of the proximal histidine H105, initiates a largely conformational change and ultimately stimulates the cyclase activity. Several models have been proposed to clarify the mechanism of NO-induced sGC activation using a variety of techniques, including mutational and truncation studies, FRET and HDX-MS *in vitro*^21^,^23^,^24^,^25^,^26^. Both these models revealed the inter-domain interactions and the conformational change upon NO binding. The heme-associated αF helix is the trigger and the PAS domain is the mediator in the NO-signaling transduction. The flexible PAS-helical linker transmits the signals from H-NOX domain to catalytic domain through helical domain. However, the detailed molecular mechanism underling NO activation of sGC is largely unknown, and the mechanism of NO-induced sGC activation *in vivo* remains unclear. Carbon oxide (CO), as another weak activator of sGC, can also stimulate sGC upon binding to the heme cofactor, but stimulation is much weaker than that of NO, which may be linked to the differences of conformational change^11^,^27^,^28^.

In this study, firstly a fluorophore FlAsH-EDT_2_, as a conformational change indicator, was introduced into the truncated and full-length sGC β1 subunits to study the NO-induced conformational change of sGC *in vitro*, which revealed that NO binding could increase the distance between the H-NOX and PAS domain and also the distance between H-NOX and coiled-coil domain. These results reflected the NO-induced allosteric effect of sGC. Secondly, FRET analysis of fluorescent proteins, which were fused to the N-/C- terminal of sGC α1 and β1 subunits, showed the conformational change from “open” to “closed” of catalytic domain. Finally, the conformational change upon NO binding was also studied *in vivo* by fluorescence lifetime imaging microscopy (FLIM)-based FRET. These results indicated the conformational change of the catalytic domain upon NO binding, which was line with the results obtained *in vitro*. The conformational allosteric effect induced the NO-signaling from H-NOX domain to the catalytic domain to ultimately stimulate the cyclase activity of sGC.

## Results

### NO-induced sGC Conformational Change *in vitro*

#### Site-specific Labeling of the sGC β1 with FlAsH-EDT_2_ and Monitoring the Distance Change by FRET

The biarsenical dye FlAsH-EDT_2_ with rather small in size can bind tightly to a small tetracysteine (TC; CCPGCC) motif and can be readily introduced by mutagenesis^29^,^30^. For the emission spectrum of FlAsH-EDT_2_ has good overlap with α and β absorbance bands of the heme, the FlAsH-EDT_2_ is a good probe to study the conformational change induced by NO binding based on the energy transfer between heme and FlAsH-EDT_2_. Previous work has been confirmed that the replacement of the residues (TC) and the FlAsH-EDT_2_ would not affect the heme microenviroment and heme binding in the sGC β1 variants [sGC β1(1-385)-^386^TC^391^, sGC β1(1-385)-^243^TC^248^ and sGC β1(1-619)-^243^TC^248^_insect_]^31^.

**Figure 1:**
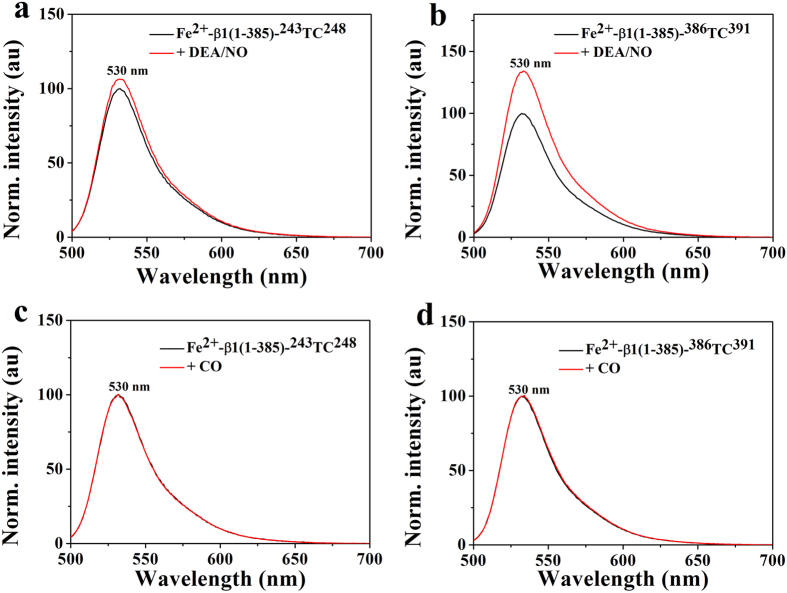
Conformational change of FlAsH-labeled sGC β1(1-385)-^243^TC^248^, and sGC β1(1-385)-^386^TC^391^ upon NO binding (**a** and **b**) or CO binding (**c** and **d**). The heme concentration was 2 μM in 20 mM HEPES, 150 mM KCl, pH 7.4.

To monitor the domain movement relative to the heme upon NO binding, the FlAsH-labeled sGC β1(1-385) truncations were anaerobically mixed with NO donator, DEA/NO, and the fluorescence change was detected as shown in Fig. 1a and b. Both the sGC β1(1-385)-^243^TC^248^ and sGC β1(1-385)-^386^TC^391^ showed an increased fluorescence. However, the conformational change degree of the sGC β1(1-385)-^386^TC^391^ with FlAsH-EDT_2_ labeled at the helical domain was much larger than that of the sGC β1(1-385)-^243^TC^248^ with FlAsH-EDT_2_ labeled at PAS domain, indicating that the distance of the helical domain moving away from the heme was much larger than the PAS domain.

Furthermore, CO-induced conformational change of FlAsH-labeled sGC β1(1-385) truncations was also monitored, the result was shown in Fig. 1c and d. The results showed no obvious change in fluorescence of FlAsH-labeled sGC β1(1-385)-^243^TC^248^ and sGC β1(1-385)-^386^TC^391^ upon CO binding, indicating that CO binding of sGC heme could not induce the conformational change. Previous work has shown that CO-bound sGC formed a six-coordinated heme and the axial His-Fe coordination bond did not break, but NO binding formed a five-coordinated heme and ruptured the Fe-N coordination bond^11^,^27^,^28^,^32^. Besides, CO has a much lower affinity and stimulates sGC activity only four-fold compared with 200-fold by NO^27^,^28^,^33^,^34^. Recently, several approaches has been applied to study the NO activation of sGC, indicating that it is the allosteric effect that activates the cyclase activity and NO-induced conformational change is responsible to transmit the NO-signaling from H-NOX to the catalytic domain^23^,^25^,^26^. Our results could explain why the CO stimulated activity was much lower than NO. Since CO binding of heme would not obviously affect sGC conformational change, thus it can be considered as a control to study the structural bases of the activation mechanism of sGC by NO. In addition, we used the mutant sGC β1(1-385)H105A-^243^TC^248^ and β1(1-385)H105A-^386^TC^391^ as another controls (see Supplementary Fig. S1), the results showed no obvious fluorescence change upon NO and CO binding. Since the sGC β1(1-385)H105A-^243^TC^248^ and β1(1-385)H105A-^386^TC^391^ could not bind heme, NO/CO binding could not induce the conformational change of sGC, which also on the other hand reflected that the small molecular NO and CO could not affect the fluorescence of the FlAsH-EDT_2_^25^.

**Figure 2:**
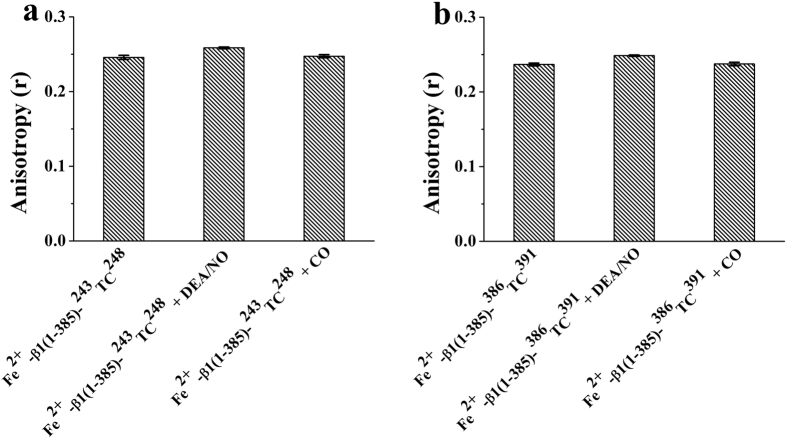
Anisotropy of FlAsH-labeled sGC β1(1-385)-^243^TC^248^ and sGC β1(1-385)-^386^TC^391^ upon NO binding (**a**) or CO binding (**b**). The heme concentration was 3 μM in 20 mM HEPES, 150 mM KCl, pH 7.4. Anisotropy data are the mean ± S.E. of two independent experiments performed in triplicate.

To rule out the possibility that the fluorophore FlAsH-EDT_2_ fluorescence change was caused by the change in the mobility or orientation of the fluorophore, the anisotropy of the FlAsH-EDT_2_ labeled sGC β1(1-385) truncations was determined before or after treatment with CO or DEA/NO, shown in Fig. 2. The anisotropy of FlAsH-EDT_2_ bound sGC β1(1-385)-^243^TC^248^ and sGC β1(1-385)-^386^TC^391^ showed no significant change upon DEA/NO or CO treatment.

**Figure 3:**
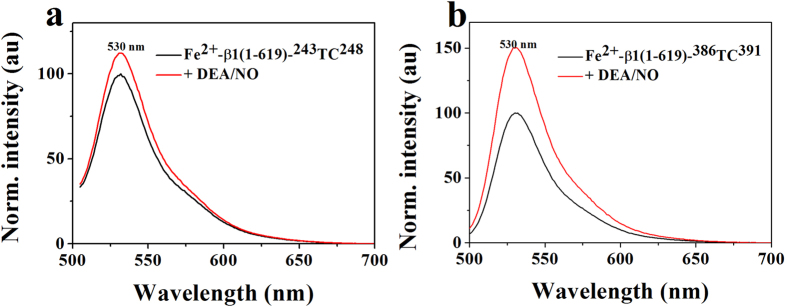
Conformational change of FlAsH-EDT_2_-labeled sGC β1(1-619)-^243^TC^248^ (**a**) and sGC β1(1-619)-^386^TC^391^ (**b**) upon NO binding. The heme concentration was 3 μM in 20 mM HEPES, 150 mM KCl, pH 7.4.

Furthermore, the full-length sGC β1(1-619)-^243^TC^248^ and β1(1-619)-^386^TC^391^ with FlAsH-EDT_2_ labeled at the PAS domain and coiled-coil domain were also applied to study the NO-induced conformational change (Fig. 3). Addition of DEA/NO to the FlAsH-labeled sGC β1(1-619)-^243^TC^248^ and β1(1-619)-^386^TC^391^, the fluorescence increased, suggesting an increased distance between heme and FlAsH-EDT_2_. As shown in Fig. 3, the conformational change trend of full-length sGC β1(1-619)-^243^TC^248^ and β1(1-619)-^386^TC^391^ was the same as that of the truncated sGC β1(1-385)-^243^TC^248^ and β1(1-385)-^386^TC^391^, but the degree was larger than that of the truncated sGC β1(1-385)-^243^TC^248^ and β1(1-385)-^386^TC^391^. It may be because that the full-length sGC contains the catalytic domain. In addition, we used the mutant sGC β1(1-619)H105A-^243^TC^248^ and β1(1-619)H105A-^386^TC^391^ as another controls (see Supplementary Fig. S2), the results showed no obvious fluorescence change upon NO and CO binding, which was the same as that of the truncated sGC β1(1-385) mutant.

#### Study on the Secondary Structure Change upon NO Binding using SRCD

**Figure 4:**
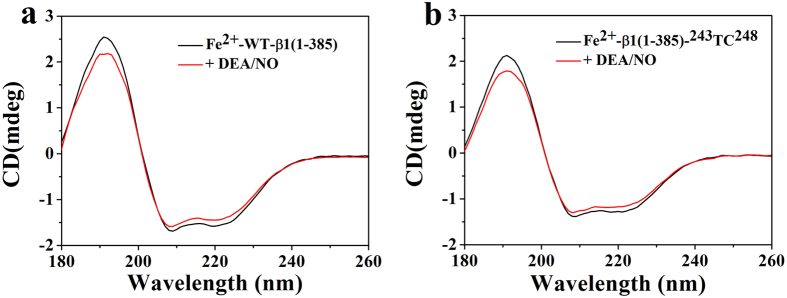
SRCD spectra in the far-UV region of sGC β1(1-385) (**a**) and sGC β1(1-385)-^243^TC^248^ (**b**) with and without DEA/NO. The heme concentration of sGC β1(1-385) and sGC β1(1-385)-^243^TC^248^ was 50 μM and 45 μM, respectively, in 10 mM HEPES, pH 7.4.

CD spectroscopy was widely used to study protein secondary structure. Synchrotron radiation circular dichroism (SRCD) had advantages relative to the conventional CD. SRCD spectroscopy with the intense light of a synchrotron beam was used to investigate the integrity of secondary structure, which had a greater sensitivity than conventional UV-CD^35^,^36^,^37^. Herein, we used SRCD to explore whether the secondary structure of sGC β1(1-385) changed upon DEA/NO binding or after the introduction of the TC motif. As shown in Fig. 4, no matter the introduction of the TC motif or the addition of the DEA/NO, the secondary structure of the sGC β1(1-385) did not be greatly disturbed (Table 1). The results indicated that NO activation of sGC did not induce the secondary structure change of sGC, which further suggested that the conformational change motioned above were indeed caused by the domain motion.

#### FRET study of fluorescent-tagged sGC variants

**Figure 5:**
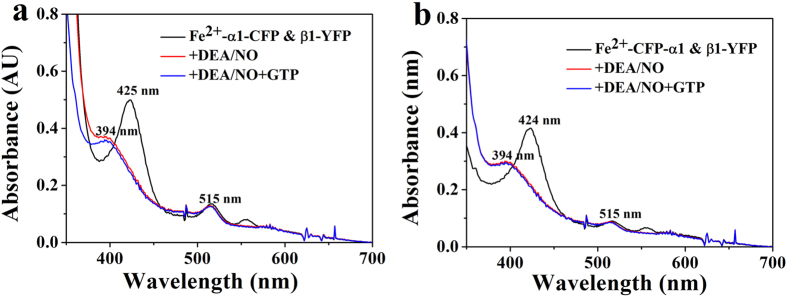
Electronic absorption spectra of sGC α1(1-690)-CFP_insect_ & β1(1-619)-YFP_insect_ (**a**) and sGC CFP-α1(1-690)_insect_ & β1(1-619)-YFP_insect_ (**b**) before and after addition of DEA/NO and the mixture of DEA/NO and substrate GTP.

Two fluorescent tagged sGC heterodimers were constructed and expressed in sf9 cells (sGC α1(1-690)-CFP_insect_ & β1(1-619)-YFP_insect_ and sGC CFP-α1(1-690)_insect_ & β1(1-619)-YFP_insect_). For sGC α1(1-690)-CFP_insect_ & β1(1-619)-YFP_insect_, the CFP and YFP were fused to the C-terminus of the α1(1-690) and β1(1-619), separately. For sGC CFP-α1(1-690)_insect_ & β1(1-619)-YFP_insect_, the CFP was fused to the N-terminus of the α1(1-690) and YFP was fused to the C-terminus of β1(1-619). Electronic absorption spectra of the two fluorescent-tagged sGC heterodimers indicated that the fused CFP or YFP did not alter the heme microenviroment and heme binding, as shown in Fig. 5. The ferrous form and NO-bound form fluorescent-tagged sGC heterodimer exhibited the similar Soret band absorbance as the wide type sGC, but the α/β band was not observed for the absorbance of YFP at about 515 nm^23^,^38^. Besides, addition of DEA/NO and the substrate GTP to the ferrous form sGC, the Soret band absorbance was similar to that of the NO-bound form sGC shown in Fig. 5. Previous work has identified that fusing the CFP and YFP to the C-terminus of sGC α or β subunit, or fusing the CFP and YFP to the N-terminus of sGC α or β subunit displayed NO and NO/YC-1 stimulated sGC activity similar to the non-tagged sGC heterodimer^23^. Thus, we used sGC α1(1-690)-CFP_insect_ & β1(1-619)-YFP_insect_ and sGC CFP-α1(1-690)_insect_ & β1(1-619)-YFP_insect_ to study the conformational change of sGC terminus upon NO activation.

**Figure 6:**
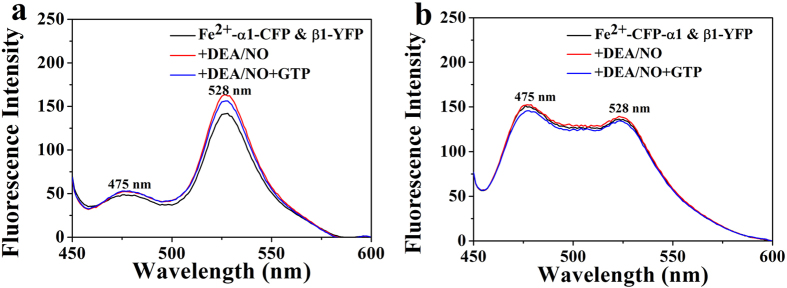
FRET analysis of purified sGC heterodimer. Fluorescent spectra of sGC α1(1-690)-CFP_insect_ & β1(1-619)-YFP_insect_ (**a**) and sGC CFP-α1(1-690) _insect_ & β1(1-619)-YFP_insect_ (**b**) at excitation wavelength of 432 nm before and after addition of DEA/NO and the mixture of DEA/NO and substrate GTP.

To examine the conformational change of the C-terminal catalytic domain of sGC under NO activation, the FRET of the sGC α1(1-690)-CFP_insect_ & β1(1-619)-YFP_insect_ was detected upon NO binding. The results shown in Fig. 6a indicated that the NO binding resulted in the increase of the FRET efficiency compared with the ferrous form sGC, suggesting that the distance of the C-terminus of sGC α1 and β1 subunit decreased. However, upon both addition of NO and the substrate GTP to sGC, the FRET efficiency showed a little decrease relative to the NO-bound sGC, but a little increase relative to the ferrous form sGC. This may be because that the GTP occupied the space of the active site in the catalytic domain and could induce the decrease of the conformational scale compared with that without GTP. Previous work has shown that NO activation could lead to conformational change of the catalytic domain from “open” state to the “closed” state^20^,^21^,^26^, which was consistent with our results.

However, the FRET study of the sGC CFP-α1(1-690)_insect_ & β1(1-619)-YFP_insect_ indicated that NO binding did not show obvious FRET efficiency change shown in Fig. 6b. Furthermore, both the NO and GTP binding also did not show obvious change in FRET efficiency. These indicated that the distance between N-terminus of α1 subunit and C-terminus of β1 subunit did not change, reflected that the N-terminus of α1 subunit did not involve in the conformational change upon NO binding and its exact role in NO activation of sGC was unclear^24^,^26^.

The result *in vitro* suggested that the conformation of discrete region changed upon NO binding, and the domain motion transmitted the NO-signaling from H-NOX domain to the catalytic domain to activate the cyclase activity. However, how the conformational changed *in vivo* was still unknown. The following work focused on studying the conformational change *in vivo* using FLIM-FRET.

### NO-induced Conformational Change *in vivo*

#### Co-immunoprecipitation Confirmed the Heterodimeric formation of sGC α1β1 and sGC α2β1 *in vivo*

**Figure 7:**
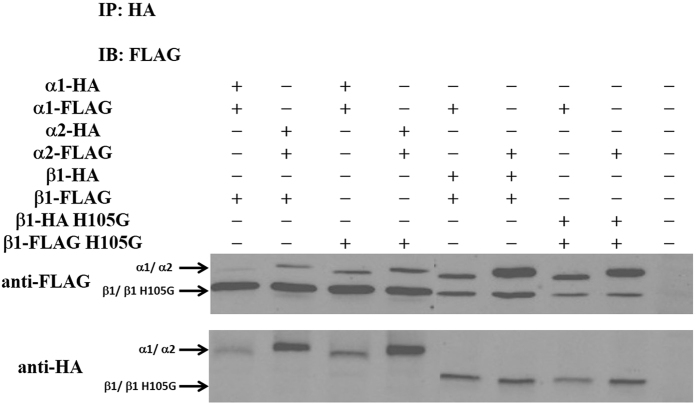
Co-immunoprecipitation analysis using transiently transfected SH-SY5Y cells expressing the wide type sGC α and β1 subunit or the sGC α and β1 mutant. Cells were co-transfected with HA- and FLAG- tagged constructs, and the HA-tagged constructs were precipitated using anti-HA antibody. Co-precipitated FLAG-tagged constructs were detected by SDS-PAGE/immunoblotting using anti-FLAG antibody (top). As a control, precipitated HA-tagged constructs were detected using anti-HA antibody (bottom). The gels were cropped and the full-length gels were shown in Supplementary Fig. S3.

Previous research has identified that only the sGC heterodimer can catalyze the conversion of GTP to cGMP and can be activated by NO. Thus, we first confirmed that the co-transfected sGC α1 or α2 and β1 constructs form the heterodimer in SH-SY5Y cells. To this end, SH-SY5Y cells were transiently co-transfected with the HA- and FLAG-tagged constructs according to the following combination: 1. α1-HA, α1-FLAG, β1-FLAG; 2. α2-HA, α2-FLAG, β1-FLAG; 3. α1-HA, α1-FLAG, β1-FLAG H105G; 4. α2-HA, α2-FLAG, β1-FLAG H105G; 5. α1-FLAG, β1-HA, β1-FLAG; 6. α2-FLAG, β1-HA, β1-FLAG; 7. α1-FLAG, β1-HA, β1-FLAG H105G; 8. α2-FLAG, β1-HA, β1-FLAG H105G. The HA-tagged constructs were precipitated using anti-HA antibodies and detected by SDS-PAGE/immunoblotting using anti-HA antibodies (Fig. 7, bottom). Co-precipitated FLAG-tagged constructs were detected using anti-FLAG antibodies (Fig. 7, top). Co-immunoprecipitation of the FLAG-tagged constructs were detectable in the case of the α1, α2, β1 and β1 H105G, indicating that the heterodimer α1β1, α2β1, α1β1 H105G and α2β1H105G were the main existing forms in SH-SY5Y cells. However, there were also a small amount of homodimer α1α1, α2α2, β1β1 and β1H105Gβ1H105G. Furthermore, the H105 mutant did not affect the formation of the heterodimer, which further identified that the H105 was not the key factor to mediate the dimerization. Previous work has shown that the PAS and helical domain play an important role in heterodimer function^13^,^15^,^17^. The Co-immunoprecipitation further indicated that sGC α and β subunits can be co-expressed well and form the heterodimer in SH-SY5Y cells, which could be used in the FLIM-FRET study.

#### NO Binding Induced sGC Conformational Change Monitored by FLIM-FRET

**Figure 8:**
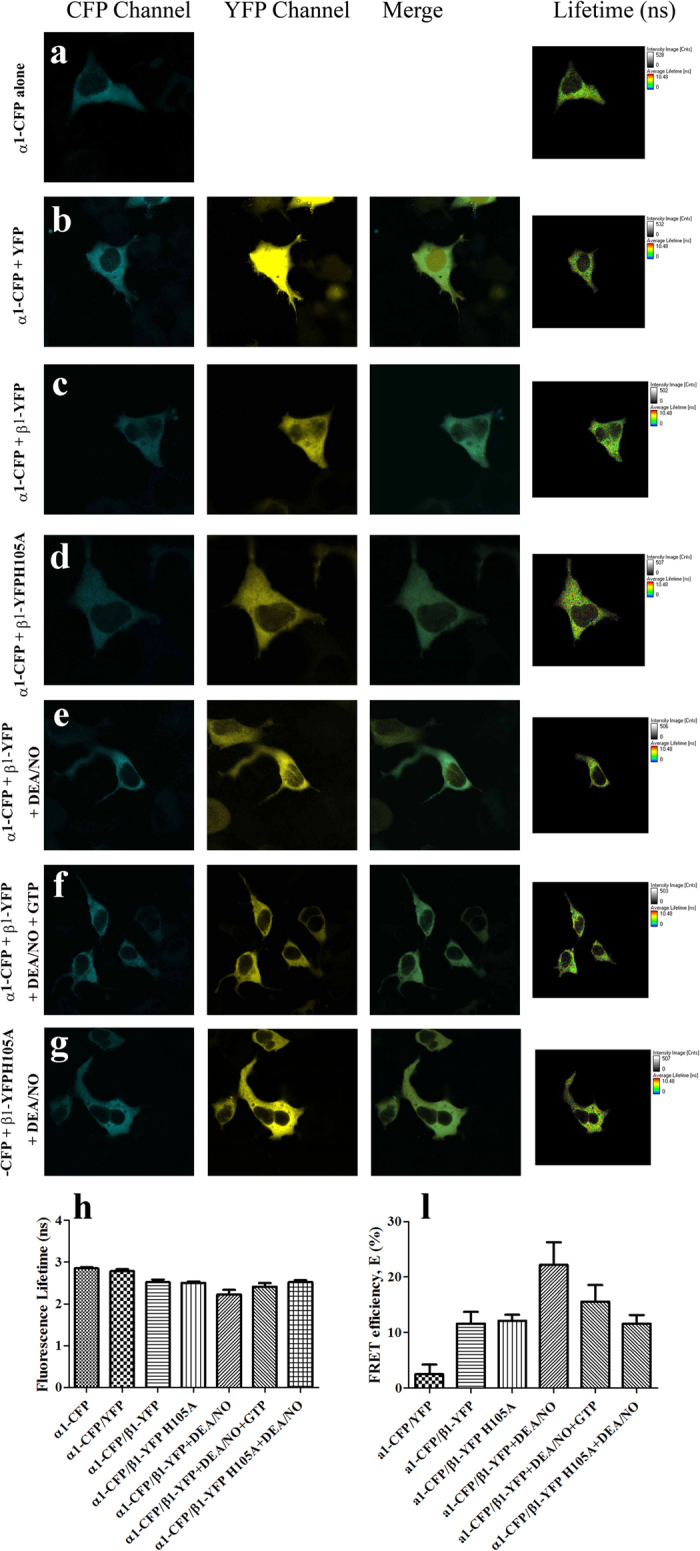
FLIM-FRET analysis in total cells. The images were taken with SH-SY5Y cells transfected with sGC β1-CFP alone (**a**), sGC β1-CFP and YFP (**b**), sGC β1-CFP and sGC β1-YFP (**c**), sGC β1-CFP and sGC β1-YFP H105A (**d**), sGC β1-CFP and sGC β1-YFP after addition of DEA/NO (**e**), sGC β1-CFP and sGC β1-YFP after addition of DEA/NO and GTP (**f**), and sGC β1-CFP and sGC β1-YFP H105A after addition of DEA/NO (**g**). Confocal images show the cellular localization of α and β subunits. The rightmost images show the lifetime images. (**h**) The lifetime values were the average lifetime through fitting the decay curve to the double exponential function from FLIM images. (**l**) FLIM-FRET efficiency (**E**) values (mean ± S.E.) were calculated based on CFP lifetime values and obtained over 5–6 cells from 4 to 5 different samples.

To further investigate the NO-induced conformational change of sGC α1β1 heterodimer *in vivo*, a FLIM-based FRET approach was applied, which allowed us to observe the interaction in live SH-SY5Y cells. The lifetime of the donor (CFP) decreased when the tagged proteins are interacting. Representative images obtained for the α1-CFP alone, negative control and experimental groups in SH-SY5Y cells were shown in Fig. 8, indicating that both sGC α1 and β1 subunits were expressed in cytoplasm, not in cell nucleus. The FLIM-FRET efficiency was calculated using CFP lifetime values and the distance between donor and acceptor was also calculated according to the equation (r = R_0_ (1/E - 1)^1/6^, R_0 CFP/YFP_ = 4.9 nm), shown in Fig. 8h and l, and Table 2. Figure 8b was the negative control with co-expression of α1-CFP and YFP in the co-transfected cells, showing no obviously change in the average mean lifetime (τ) relative to the expression of α1-CFP alone in the cells. Co-expression of α1-CFP and β1-YFP in the SH-SY5Y cells resulted in the decreased fluorescence lifetime compared with the α1-CFP alone, indicating that the sGC α1 interacted with sGC β1. Besides, the lifetime of α1-CFP co-transfected with the mutant β1-YFP H105A into the SH-SY5Y cells also decreased, identical to the wide-type with the same FLIM-FRET efficiency (Fig. 8c,d,h and l). The results indicated that the mutant H105A affect neither the interaction between sGC α1 and β1, nor the distance between C-terminus of the sGC α1 and β1. However, addition of DEA/NO to live SH-SY5Y cells with the co-expressed α1-CFP and β1-YFP, the lifetime of the donor α1-CFP decreased relative to that without DEA/NO. The results indicated that NO binding increased the FLIM-FRET efficiency and decreased the distance between C-terminus of the sGC α1 and β1 about 8.4 Å shown in Fig. 8(c,e,h and l). Moreover, upon adding both NO and GTP to sGC in the co-expressed α1-CFP and β1-YFP in live SH-SY5Y cells, the lifetime of the donor α1-CFP increased compared with that only with DEA/NO, but also decreased compared with that without DEA/NO and GTP. The distances decreased about 3.8 Å relative to that in the cells only co-transfected with α1-CFP and β1-YFP, as shown in Fig. 8(c,f,h and l). The results also indicated the conformational change of the catalytic domain upon activation by NO *in vivo*, which further was identified with the results in *vitro* as shown in Fig. 6a. Besides, addition of DEA/NO to live SH-SY5Y cells with the co-expressed α1-CFP and β1-YFP H105A, the lifetime did not change obviously compared with that without adding DEA/NO shown in Fig. 8(d,g,h and l), indicating that the mutant H105A could not induced the conformational change of the catalytic domain, and also reflecting that the small molecular NO could not affect the lifetime of the fluorescent proteins *in vivo*. The results were consistent with that obtained *in vitro*.

**Figure 9:**
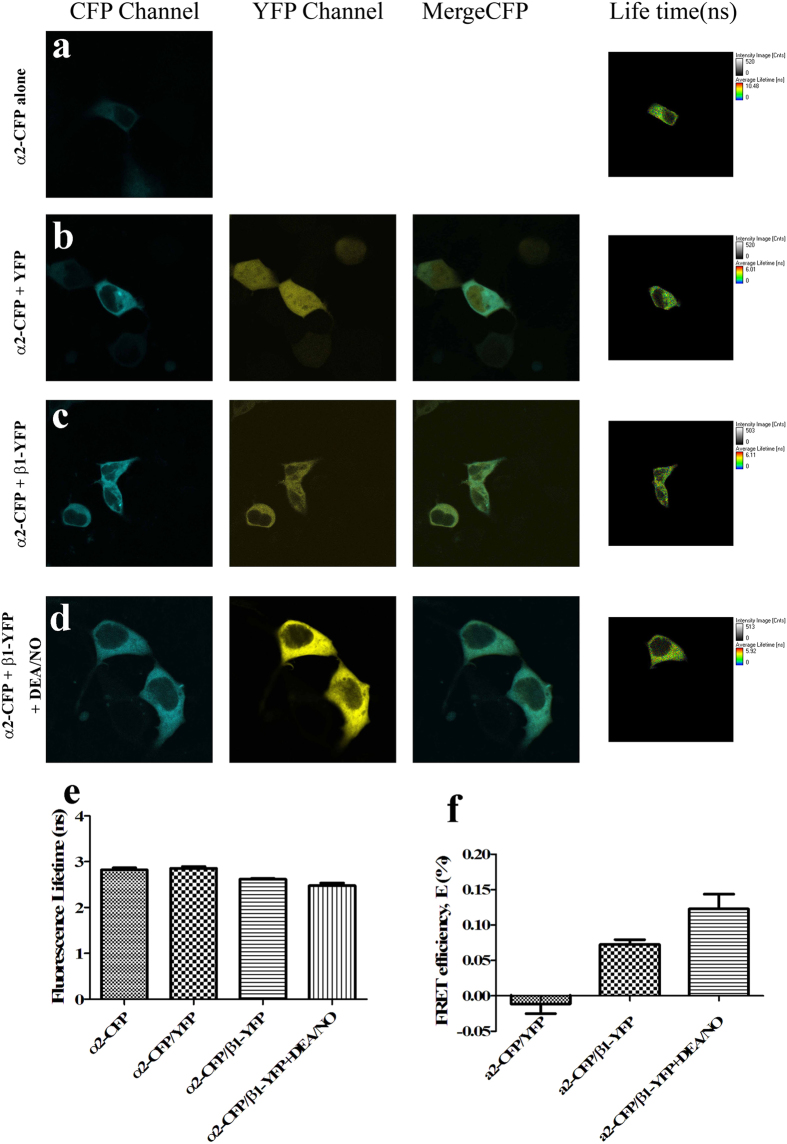
FLIM-FRET analysis in total cells. The images were taken with SH-SY5Y cells transfected with sGC α2-CFP alone (**a**), sGC α2-CFP and YFP (**b**), sGC α2-CFP and sGC β1-YFP (**c**), and sGC α2-CFP and sGC β1-YFP after addition of DEA/NO (**d**). Confocal images show the cellular localization of α and β subunits. The rightmost images show the lifetime images. (**e**) The lifetime values were the average lifetime through fitting the decay curve to the double exponential function from FLIM images. (**f**) FLIM-FRET efficiency *(E)* values (mean ± S.E.) were calculated based on CFP lifetime values and obtained over 5–6 cells from 4 to 5 different samples.

To further identify that the NO binding really causes the conformational change of the C-terminus of the sGC catalytic domain, the FLIM-FRET experiments in the live SH-SY5Y cells co-transfected with sGC α2-CFP and sGC β1-YFP were also performed and the results are shown in Fig. 9. The negative control with co-expression with α2-CFP and YFP showed no obviously change in the lifetime of the donor α2-CFP. The addition of DEA/NO to the co-expressed α2-CFP and β1-YFP live SH-SY5Y cells, the lifetime of the donor α2-CFP also decreased relative to that without DEA/NO. This indicated that NO binding increased the FLIM-FRET efficiency and decreased the distance between C-terminus of the sGC α2 and β1 about 6.8 Å, as shown in Fig. 9 and Table 3. The results were identical to that of the cells co-transfected with α1-CFP and β1-YFP. Thus, we could speculate that no matter the heterodimer α1β1 or the α2β1, the NO-induced conformational change was the similar in which the decreased distance between C-terminus of sGC catalytic domain was about 7–8 Å *in vivo*. These results are consistent with what previous reported that the catalytic domain of the sGC can change from “open” state to the “closed” state^20^,^21^,^26^.

## Discussions

The molecular mechanism underlying NO activation of sGC is a central question in NO-sGC-cGMP signaling. To understand the NO-signaling molecular mechanism mediated by sGC, several studies *in vitro* have been reported. Behrends *et al*. identified the sGC domains organization using the fluorescent proteins through monitoring the FRET change *in vitro*, supporting the “trans” regulation of sGC activity by the H-NOX domains proposed by Marletta^19^,^23^. Recently, Behrends and co-workers also studied the conformational change of sGC upon activation by NO through monitoring the energy transfer between the endogenous tryptophan residues and the substrate analogue 2′-Mant-3′-dGTP *in vitro*, suggesting that the respective domains act as a pair of tongs forcing the catalytic domain into the NO activated conformation^25^. In addition, very recently Marletta *et al*. studied the NO-induced conformational change using HDX-MS and indicated the domains interaction upon NO binding^26^. Although great progress has been made on the NO-induced conformational change of sGC *in vitro* as described above, the exact interaction of the inter-domains of sGC *in vitro* was unclear and the NO-induced conformational change in living cells remained unknown. Therefore, in this study, biarsenical fluorophore FlAsH-EDT_2_ and fluorescent proteins (CFP and YFP) were employed to study the NO-induced sGC activation mechanism *in vitro* and *in vivo*. FlAsH-EDT_2_ labeling experiments *in vitro* revealed that NO binding to the H-NOX domain of sGC increased the distance between H-NOX and PAS domain and the distance between H-NOX and coiled-coil domain. FRET analysis of fluorescent proteins *in vitro*, which were fused to the N-/C- terminal of the α1 and β1 subunits of sGC, exhibited the conformational change from “open” to “closed” state of sGC catalytic domain. In addition, the NO-induced conformational change of sGC was firstly investigated *in vivo* through fluorescence lifetime imaging microscopy FLIM-based FRET. The results also indicated the conformational change of the catalytic domain from the “open” to the “closed” state with the distance decreased about 8 Å upon NO binding, which was in line with the results *in vitro*. NO binding to H-NOX domain induced the allosteric effect of sGC to trigger the NO-signaling from H-NOX domain to the catalytic domain and ultimately stimulates the cyclase activity of sGC.

NO binding to the heme resulted in the rupture of the Fe^2+^-N coordination and released the heme-associated αF helix^24^,^39^,^40^. The displacement of the αF helix was also observed in *Shewanella oneidensis* H-NOX variant without the His ligand^41^. Besides, the replacement of the heme with the small molecular sGC activator BAY 58-2667 caused a rotation of the αF helix away from the heme pocket for removing the inhibitory of the His-heme coordination bond in the *Nostoc.sp. PCC 7120 H-NOX*^42^. Recent HDX-MS analysis has revealed the increased exchange rate in the heme-associated αF helix upon NO binding and implicated that the heme-associated αF helix was a focal point of the NO-induced conformational change^26^.

Interestingly, upon NO binding, both the PAS and coiled-coil domain of the truncated sGC β1(1-385) shifted away from the heme through monitoring the fluorescence of the labeled FlAsH-EDT_2_, which was shown in Fig. 1a and b. However, the degree of the conformational change of PAS domain was smaller than that of the coiled-coil domain. Previous work revealed the direct interaction between heme-associated αF helix and PAS domain, and the conformational change recognized by the abutting PAS domain^21^,^26^. Besides, the PAS domain was a key mediator to transmit the conformational signals from the H-NOX domain to the catalytic domain. Thus, it is speculated that the heme-associated αF helix motion initiated the movement of the PAS domain (Fig. 1a). Recent single-particle EM results have shown that the high-order architecture was consisted of two rigid domains: the catalytic domain and the clustered H-NOX and PAS domain, connected by the coiled-coil domain at two flexible hinge points^20^. The PAS-helix linker and the helix-catalytic linker played an important role in transducing the conformational signals^26^. The PAS-helix linker might induce the helix domain movement away from the heme (Fig. 1b). Furthermore, prior work indicated that the direct interaction between the H-NOX domain and catalytic domain repress the sGC activity, which was supported by a FRET study that revealed close proximity between N-terminus and C-terminus of sGC^19^,^23^. Recent HDX-MS analysis revealed the releasing inhibitory between H-NOX and catalytic domain caused by the motion of the PAS-helix junction^26^. Thus, the movement of H-NOX domain away from the catalytic domain could explain why the conformational degree of coiled-coil domain was larger than the PAS domain relative to the heme (Fig. 3).

The FRET study using the fluorescent fusion proteins *in vitro* indicated that the distance between the C-terminus of the sGC α and β subunit decreased upon NO activation. Furthermore, the result obtained *in vivo* through FLIM-FRET study in live SH-SY5Y cells also showed the decreased distance between the C-terminus of the sGC α and β subunit. Although the change from the “open” to the “closed” was just the change of the conformation of the C-terminus of the sGC heterodimer, it can indirectly reflect the NO-signaling transduction from the H-NOX domain to the catalytic domain, which may involve the discrete domain movement of sGC. Recent HDX-MS study revealed that the catalytic domain changed from the inactive “open” state to the active “closed” state upon NO activation^26^.

**Figure 10:**
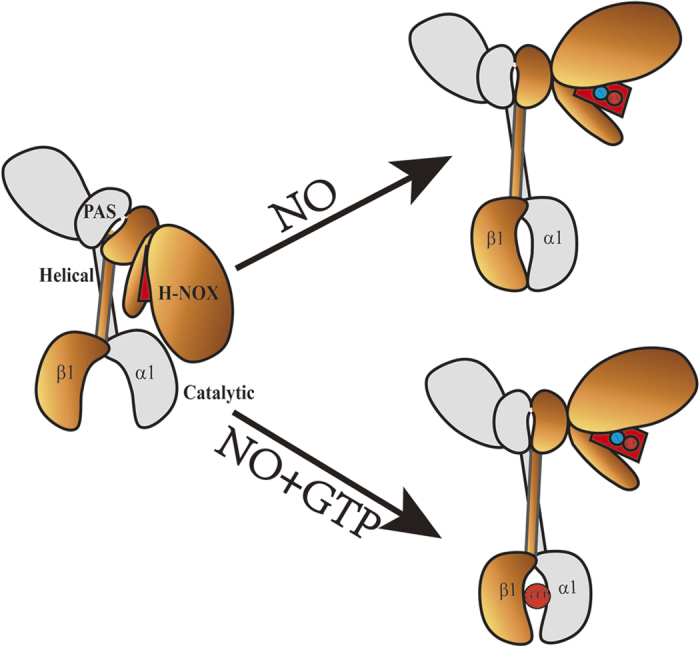
A proposed NO-Induced activation mechanism of sGC.

Based on the results obtained in *vitro* and in *vivo*, we proposed a most likely molecular mechanism underlying NO activation of sGC based on the high-order domain architecture from single-particle EM shown in Fig. 10, and various labeled proteins in relation to the sGC three dimensional model were shown in Supplementary Fig. S4. NO binding released the heme-associated αF and induced the PAS and coiled-coil domain moving away from the H-NOX domain, and ultimately led the catalytic domain change from the “open” state to the “closed” state to activate the cyclase activity of sGC. In addition, the GTP could occupy the space of the active site in the catalytic domain and induce the decrease of the conformational scale. Understanding the molecular mechanism underlying NO activation of sGC would be helpful to the development of therapeutics targeting cGMP-dependent physiological processes.

In conclusion, the NO-induced conformational change was studied using the fluorophore FlAsH-EDT2 and fluorescent proteins *in vitro*, which indicated that the NO binding to the H-NOX domain of sGC increased the distance between H-NOX and PAS domain, and led to the conformation change of the catalytic domain from the “open” to “closed”. And most of all, we firstly studied the conformational change of sGC upon NO binding *in vivo* based on the lifetime imaging microscopy, which indicated the conformational change of the catalytic domain from “open” to “closed”. We expect that the NO-induced activation mechanism of sGC will promote the development of therapeutics targeting cGMP-dependent physiological processes.

## Methods

### Materials

FlAsH-EDT_2_ was purchased from Toronto research chemicals Inc., and the transfection reagents Lipofectamine^TM^ 2000 were from Invitrogen. The monoclonal mouse anti-FLAG antibody was from Abcam and the monoclonal rabbit anti-HA was from Sigma. Pierce^®^ Classic IP Kit (26146) was from Thermo Scientific. KOD-Plus-Mutagenesis Kit was from TOYOBO. The plasmid purification kit (12362), nickel nitrilotriacetic acid (Ni-NTA) resin and Sephadex G-25 resin were purchased from QIAGEN. DEAE Sepharose^TM^ Fast Flow was purchased from GE Healthcare Bio-Science. SH-SY5Y cells were from the National Laboratory of Neurobiology of Fudan University. All other reagents were of analytic grade.

### Plasmid Constructs

**Figure 11:**
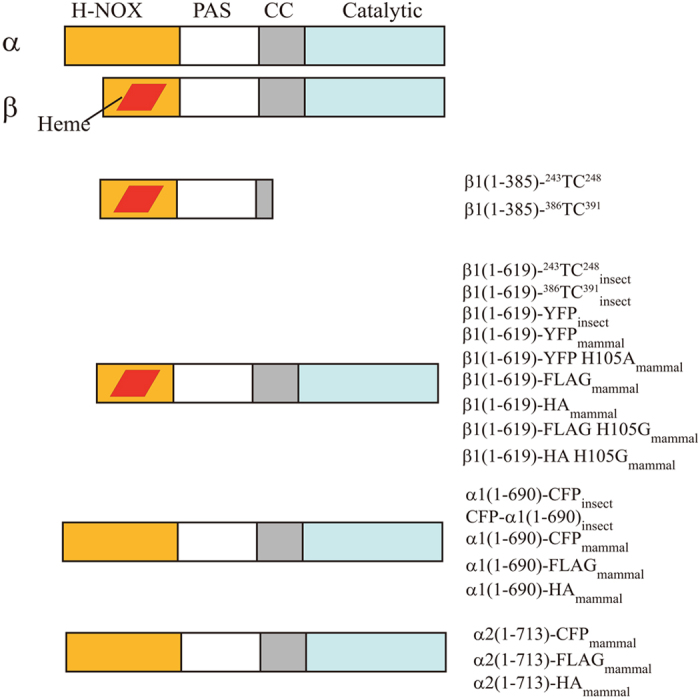
The domain architectures of sGC and schematic representations of sGC variants constructs used in this study.

The plasmid constructs used in this study were schematically shown in Fig. 11. The plasmids [sGC β1(1-385)-^386^TC^391^, sGC β1(1-385)-^243^TC^248^ and sGC β1(1-619)-^243^TC^248^_insect_], which were introduced with a small tetracysteine (TC: CCPGCC), have been constructed as described before^31^. The construction of the plasmid of sGC β1(1-619)-^386^TC^391^_insect_ was the same as the sGC β1(1-619)-^243^TC^248^_insect_. For the FRET experiment *in vitro*, the plasmids of sGC β1(1-619)-YFP_insect_, sGC α1(1-690)-CFP_insect_ and sGC CFP-α1(1-690)_insect_ were constructed as described below. For the sGC β1(1-619)-YFP_insect_, the YFP was C-terminally tagged to the pBAC-1-sGC β1(1-619) using restriction enzymes *N*otI and *X*hoI. For the sGC α1(1-690)-CFP_insect_ and sGC CFP-α1(1-690)_insect_, the constructions contained a two-step procedure: firstly inserting the α1(1-690) using the restriction enzymes *H*indIII and *N*otI of the vector pBAC-1, and secondly subcloning CFP in the C-terminus using the restriction enzymes *N*otI and *X*hoI or in the N-terminus using the restriction enzymes *B*amHI and *H*indIII. For the FLIM-FRET experiment *in vivo*, the plasmids were all constructed into the vector pcDNA3.1(−). Firstly, we constructed the β1(1-619), α1(1-690) and α2(1-713) into the vector pcDNA3.1(−), and secondly YFP and CFP were C-terminally fused to the β1(1-619), α1(1-690) and α2(1-713) [β1(1-619)-YFP_mammal_, α1(1-690)-CFP_mammal_ and α2(1-713)-CFP_mammal_], respectively. In addition, the His105 in the β1 subunit was mutated to alanine [β1(1-619)-YFP H105A_mammal_]. The pcDNA3.1(−)-CFP and pcDNA3.1(−)-YFP were used as a control. For the co-immunoprecipitation experiment, the FLAG/HA tag was C-terminally fused to the β1(1-619), β1(1-619) H105G, α1(1-690) and α2(1-713), respectively, as shown in Fig. 11.

### Expression and Purification of sGC variants

The truncated sGC β1(1-385) variants [sGC β1(1-385)-^386^TC^391^ and sGC β1(1-385)-^243^TC^248^] were expressed in *E. coli*. and purified as described before^31^. The protein concentration was estimated by the Soret peak using the pyridinehemochromagen assay. All the purified processes were performed in a glove-box under high pure nitrogen. The full-length sGC variants were all expressed in insect sf9 cells. The recombinant baculo-viruses of respective proteins [sGC β1(1-619)-^243^TC^248^_insect_, sGC β1(1-619)-^386^TC^391^_insect_, sGC β1(1-619)-YFP_insect_, sGC α1(1-690)-CFP_insect_ and sGC CFP-α1(1-690)_insect_] were generated according to the BAC-TO-BAC^TM^ System. Sf9 cells were cultured in SF900II medium supplemented with 1% penicillin/streptomycin at 27 °C. To generate sGC β1(1-619)-^243^TC^248^_insect_ or sGC β1(1-619)-^386^TC^391^_insect_, sf9 cells were transfected with sGC β1(1-619)-^243^TC^248^_insect_ viruses or sGC β1(1-619)-^386^TC^391^_insect_ viruses. To generate recombinant sGC heterodimer carrying various fluorescent-tagged proteins, sf9 cells were co-transfected with α1- and β1-expression viruses. For example, to generate sGC α1(1-690)-CFP_insect_ & β1(1-619)-YFP_insect_, Sf9 cells were co-transfected with sGC α1(1-690)-CFP_insect_ and β1(1-619)-YFP_insect_ viruses. The following purification processes were performed in a glove-box under high pure nitrogen as described before^31^. The recombinant proteins expressed in sf9 cells were obtained without heme group, and the heme reconstitution was performed as previously^5^. All the purification processes and heme reconstitution were performed in a glove-box under high pure nitrogen.

### sGC Proteins Labeling with FlAsH-EDT_2_ *in vitro*

The FlAsH-EDT_2,_ which can bind tightly to a small tetracysteine (TC: CCPGCC), was readily introduced into different position of sGC proteins. The procedure of the sGC variants [sGC β1(1-385)-^243^TC^248^, sGC β1(1-385)-^386^TC^391^, the full-length sGC β1(1-619)-^243^TC^248^_insect_ and sGC β1(1-619)-^386^TC^391^_insect_] were labeled with the FlAsH-EDT_2_ as described before^31^. In brief, the sGC proteins were reduced with 1 mM TCEP and then allowed to stand at room temperature for 2-3 h. The mixtures were diluted to 20 μM to incubate with 1.5 equivalents of FlAsH-EDT_2_ in buffer A (20 mM HEPES, 150 mM KCl, 1 mM TCEP, pH 7.4) for 3 h at room temperature in dark, and then applied to PD10 column to remove the excessive reagents in buffer B (20 mM HEPES, 150 mM KCl, pH 7.4). All of the processes were operated in a glove box under high pure nitrogen.

### UV-visible Spectroscopy

Electronic Absorbent spectra of all the purified sGC variants in buffer B (20 mM HEPES, 150 mM KCl, pH 7.4), were recorded in anaerobic cuvette on an HP8453 UV-visible spectrophotometer. The preparation of the ferrous protein samples were as described before^31^. The heme concentration was 2 μM or 3 μM [ε_431nm_ = 120 mM^−1^ cm^−1^ for β1(1-385), ε_424nm_ = 110 mM^−1^cm^−1^ for β1(1-619)]. The NO/CO-bound sGC proteins were produced by adding NO donator (DEA/NO) or bubbling with CO for 5 min.

### FRET Measurements of sGC Proteins *in vitro*

The spectrofluorometric study was carried out in anaerobic cuvette with a Varian spectrofluorometer. The heme group can quench the FlAsH-EDT_2_ through the Förster resonance energy transfer (FRET) for the good overlap with α and β absorbance bands of sGC heme group. Therefore, for the pair of FlAsH-EDT_2_ and heme, the excitation was at 485 nm and emission spectra were recorded from 500 nm to 700 nm. In addition, for the pair of CFP and YFP, the excitation was at 432 nm and emission spectra were measured from 450 nm to 600 nm.

### Fluorescence Anisotropy of sGC Proteins Labeled with FlAsH-EDT_2_

The fluorescence anisotropy was measured as described^31^. Samples were excited at 485 nm. For determining the anisotropy of NO/CO-bound sGC proteins, samples were mixed and incubated for several minutes at room temperature.

### Synchrotron Radiation Circular Dichroism Spectroscopy (SRCD)

The SRCD spectra of the truncated wide type sGC β1(1-385) and its variant β1(1-385)-^243^TC^248^ were recorded from 190 nm to 270 nm at Beijing Synchrotron Radiation Facility (BSRF) beam line 4B8 in the Institute of High Energy Physics, Chinese Academy of Science. The cell chamber was 0.1 mm and filled with N_2_. The temperature was kept at 4 °C. The protein buffer was buffer C (10 mM HEPES, pH 7.4).

### Cell Culture and Transfection

The SH-SY5Y cells were cultured in Dulbecco’s modified Eagle’s medium (DMEM) containing 10% fetal calf serum, at 37 °C under 5% CO_2_. Transfection of the cells with Lipofectamine^TM^ 2000 was carried out according to the supplier’s recommendations 24 h after seeding of cells. Cells were used 44–45 h later after transfection.

### Co-Immunoprecipitation

The FLAG and HA tagged plasmids were used for the Co-immunoprecipitaton. Co-immunoprecipitaton was carried out using Pierce^®^ Classic IP Kit (26146) according to the supplier’s protocol. In brief, SH-SY5Y cells (3 × 10^6^) were grown on 100-mm dishes and transiently co-transfected with plasmids using Lipofectamine^TM^ 2000 according to the supplier’s recommendations. Cells were cultivated for 48 h, washed twice with PBS buffer (pH 74), and lysed for 5 min with 750 μl ice cold IP Lysis/Wash Buffer. Insoluble debris was discarded by centrifugation (~13,000 × g, 10 min). The supernatant was supplemented with monoclonal rabbit anti-HA antibody and incubated overnight at 4 °C to form the immune complex. Then the antibody/lysate sample was supplemented with Protein A/G Plus Agarose and incubated with gentle end-over-end for 1 h. The resin was washed three times with IP Lysis/Wash Buffer and once with 1×Conditioning Buffer, and then the FLAG-tagged constructs were precipitated. The immunoprecipitated proteins were analyzed *via* Western blot analysis.

### Fluorescence Lifetime Image Microscopy (FLIM)-FRET

FLIM-FRET experiments were performed on Leica TCS SP8 equipped with SMD detector. SH-SY5Y cells transiently expressing the donors sGC α1(1-690)-CFP_mammal_ or sGC α2(1-713)-CFP_mammal_ alone or with YFP (as a control experiment), sGC β1(1-619)-YFP_mammal_ or sGC β1(1-619)-YFP H105A_mammal_ (acceptor) were grown on a Confocal Dish (Coverglass-Bottom Dish), and visualized using an upright fluorescence microscope, and observed through a 25× water immersion objective at room temperature. Channel pictures were taken prior to the recording of the FLIM pictures in order to estimate expression of the CFP- and YFP-tagged constructs as described above. The laser diode emitting at 440 nm were used for excitation and the emission was monitored using 450–490 nm detection filter. Lifetime images were analyzed by fitting a bi-exponential model equation to the fluorescence decay in every pixel of the image. To include all possible populations and conformations of the CFP donor (those that do and those that eventually do not do FRET), the fluorescence lifetime displayed in FLIM images or lifetime histograms corresponds to the average fluorescence lifetime calculated as the amplitude weighted mean value using the data from the bi-exponential fit. FRET was analyzed by monitoring the fluorescence lifetime of the donor in the presence and absence of acceptor and calculated according to formula E = 1 − τ_FRET_/τ_CFP_^43^,^44^.

## Additional Information

**How to cite this article**: Pan, J. *et al*. Probing the Molecular Mechanism of Human Soluble Guanylate Cyclase Activation by NO *in vitro* and *in vivo. Sci. Rep.* **7**, 43112; doi: 10.1038/srep43112 (2017).

**Publisher's note:** Springer Nature remains neutral with regard to jurisdictional claims in published maps and institutional affiliations.

## Supplementary Material

Supplementary Information

## Figures and Tables

**Table 1:** The calculated secondary structure composition of sGC β1(1-385) and sGC β1(1-385)-^243^TC^248^ with and without DEA/NO.

	α-Helix	β-sheet	Turns	Unordered
sGC β1(1-385)	0.42	0.29	0.07	0.22
sGC β1(1-385) + DEA/NO	0.43	0.27	0.08	0.22
sGC β1(1-385)-^243^TC^248^	0.41	0.30	0.07	0.22
sGC β1(1-385)-^243^TC^248^ + DEA/NO	0.39	0.30	0.07	0.23

**Table 2:** FLIM-FRET efficiency and distance distribution of CFP and YFP-tagged sGC heterodimers co-expressed in SH-SY5Y cells.

	Efficiency (%)	Distance (Å)
sGC α1-CFP/YFP	2.5 ± 1.4	93.8 ± 10.4
sGC α1-CFP&β1-YFP	11.6 ± 1.8	68.9 ± 2.2
sGC α1-CFP&β1-YFP H105A	12.0 ± 1.0	68.3 ± 1.2
sGC α1-CFP&β1-YFP + DEA/NO	22.2 ± 3.5	60.5 ± 2.1
sGC α1-CFP&β1-YFP + DEA/NO + GTP	15.6 ± 2.4	65.1 ± 2.1
sGC α1-CFP&β1-YFP H105A + DEA/NO	11.6 ± 1.3	68.8 ± 1.5

**Table 3:** FLIM-FRET efficiency and distance distribution of CFP and YFP-tagged sGC heterodimers co-expressed in SH-SY5Y cells.

	Efficiency (%)	Distance (Å)
sGC α2-CFP/YFP	−1.1 ± 0.01	n/a
sGC α2-CFP&β1-YFP	7.3 ± 0.67	75.0 ± 1.1
sGC α2-CFP&β1-YFP + DEA/NO	13.6 ± 1.5	68.2 ± 2.2
