# Supplementary material for: Probing the Molecular Mechanism of Human Soluble Guanylate Cyclase Activation by NO in vitro and in vivo
Source: Sci Rep. 2017 Feb 23;7:43112. doi: 10.1038/srep43112 (PMC5322342; doi:10.1038/srep43112)

Probing the Molecular Mechanism of Human Soluble Guanylate Cyclase Activation by NO in *vitro* and in *vivo*

Jie Pan[1], Hong Yuan[1], Xiaoxue Zhang[1], Huijuan Zhang[1], Qiming Xu[2], Yajun Zhou[1], Li Tan[3], Shingo Nagawa[3], Zhong-Xian Huang[1] and Xiangshi Tan[*1,2]

[1]Department of Chemistry & Shanghai Key laboratory of Chemical Biology for Protein Research, Fudan University, Shanghai 200433, China
[2]Institutes of Biomedical Sciences, Fudan University, Shanghai 200433, China
[3]Shanghai Center for Plant Stress Biology, Shanghai Institutes for Biological Sciences, Chinese Academy of Sciences, Shanghai 200433, China

*Correspondence to xstan@fudan.edu.cn

Fig.S1 Conformational change of FlAsH-labeled sGC β1(1-385)H105A-^{243}TC^{248} (a), and sGC β1(1-385)H105A-^{386}TC^{391} (b) upon NO binding or CO binding. The protein was 2 μM in 20 mM HEPES, 150 mM KCl, pH 7.4.

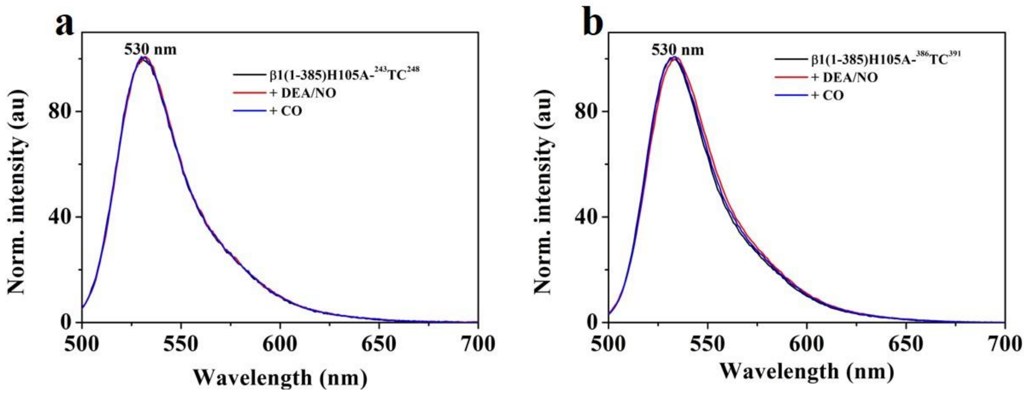

Fig.S2 Conformational change of FlAsH-labeled sGC β1(1-619)H105A-^{243}TC^{248} (a), and sGC β1(1-619)H105A-^{386}TC^{391} (b) upon NO binding or CO binding. The protein was 3 μM in 20 mM HEPES, 150 mM KCl, pH 7.4.

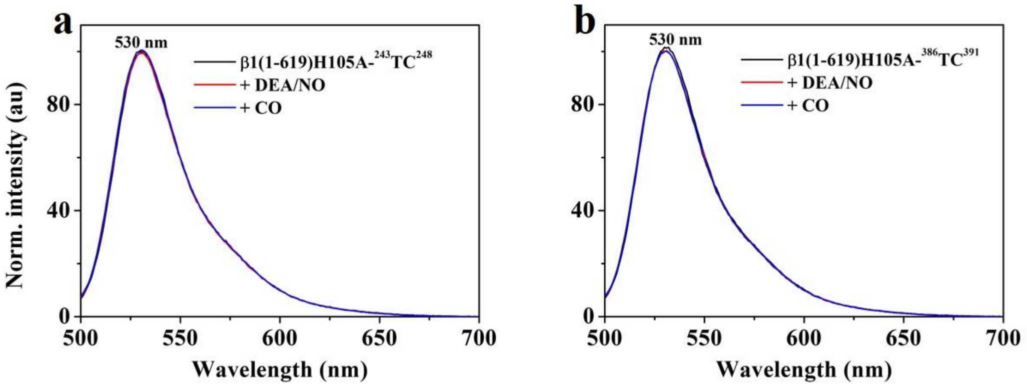

Fig.S3 The full-length gel of the western-blot shown in Figure 7.

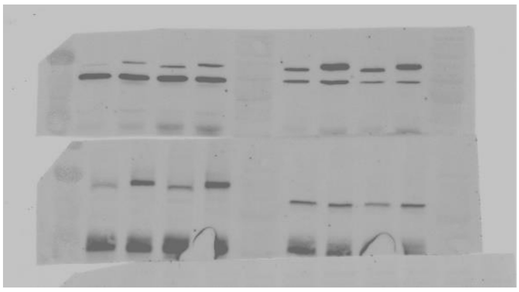

Fig.S4 The model summarizing various labeled proteins in relation to the sGC three dimensional models. FlAsH-EDT$_2$ labeled to the PAS domain (A) and coil-coiled domain (B); CFP and YFP fused to C-terminus of α1subunit and β1subunit (C); CFP fused to the N-terminus of α1subunit and YFP fused to the C-terminus of β1subunit (D).

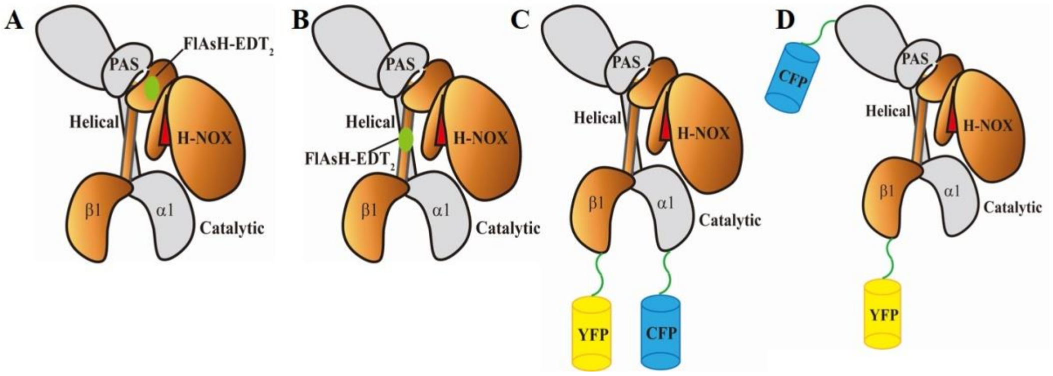

Supplement: Supplementary Information [file srep43112-s1.pdf]
